# Sex-specific associations between adolescent categories of BMI with cardiovascular and non-cardiovascular mortality in midlife

**DOI:** 10.1186/s12933-018-0727-7

**Published:** 2018-06-05

**Authors:** Ariel Furer, Arnon Afek, Omri Orr, Liron Gershovitz, Moran Landau Rabbi, Estela Derazne, Orit Pinhas-Hamiel, Noam Fink, Adi Leiba, Amir Tirosh, Jeremy D. Kark, Gilad Twig

**Affiliations:** 1 The Israel Defense Forces Medical Corps, Tel Hashomer, Ramat Gan, Israel; 2 The Sackler School of Medicine, Tel Aviv University, Tel Aviv, Israel; 3 Pediatric Endocrine and Diabetes Unit, Edmond and Lily Safra Children’s Hospital, Sheba Medical Center, Ramat-Gan, Israel; 4 The Dr. Pinchas Bornstein Talpiot Medical Leadership Program, Sheba Medical Center, Ramat-Gan, Israel; 5 Institute of Endocrinology, Sheba Medical Center, Tel Hashomer, Ramat-Gan, Israel; 6 The Division of Endocrinology, Diabetes and Hypertension, Brigham and Women’s Hospital, Harvard Medical School, Boston, MA USA; 7 Hebrew University-Hadassah School of Public Health and Community Medicine, Ein Kerem, Jerusalem, Israel; 8 Department of Medicine, Sheba Medical Center, Tel Hashomer, Ramat-Gan, Israel

**Keywords:** Adolescence, Body mass index, Cohort study, Obesity, Cardiovascular death, Sex, Women, Underweight

## Abstract

**Context:**

Most studies linking long-term consequences of adolescent underweight and obesity are limited to men.

**Objective:**

To assess the sex-specific association of adolescent BMI with cardiovascular- and non-cardiovascular-related mortality in young adulthood and midlife.

**Setting:**

A nationwide cohort.

**Participants:**

927,868 women, 1,366,271 men.

**Interventions:**

Medical examination data at age 17, including BMI, were linked to the national death registry.

**Main outcomes:**

Death attributed to cardiovascular (CVD) and non-CVD causes.

**Results:**

During 17,346,230 women-years and 28,367,431 men-years of follow-up, there were 451 and 3208 CVD deaths, respectively, and 6235 and 22,223 non-CVD deaths, respectively. Compared to low-normal BMI (18.5–22.0 kg/m^2^), underweight women had a lower adjusted risk for CVD mortality (Cox hazard ratio (HR) = 0.68; 95% CI 0.46–0.98) in contrast to underweight men (HR = 0.99; 0.88–1.13). The latter were at higher risk for non-CVD mortality (HR = 1.04; 1.00–1.09), unlike underweight women (HR = 1.01; 0.93–1.10). Findings, which persisted when the study sample was limited to those with unimpaired health, were accentuated for the obese with ≥ 30 years follow-up. Both sexes exhibited similarly higher risk estimates already in the high-normal BMI range (22.0 ≤ BMI < 25.0 kg/m^2^) with overall no interaction between sex and BMI (p = 0.62). Adjusted spline models suggested lower BMI values for minimal mortality risk among women (16.8 and 18.2 kg/m^2^) than men (18.8 and 20.0 kg/m^2^), for CVD and non-CVD death, respectively.

**Conclusions:**

Underweight adolescent females have favorable cardiovascular outcomes in adulthood. Otherwise the risk patterns were similar between the sexes. The optimal BMI value for women and men with respect to future CVD outcomes is within or below the currently accepted low-normal BMI range.

**Electronic supplementary material:**

The online version of this article (10.1186/s12933-018-0727-7) contains supplementary material, which is available to authorized users.

## Introduction

Cardiovascular disease remains the leading cause of death in the Western world [1, 2]. Over the last few decades, the prevalence of obesity has risen worldwide to as high as 20% of the adolescent population in the US [3]. There is increasing evidence for the link between adolescent overweight and obesity and cardiovascular and all-cause mortality [4–6].

Many of the studies included only men [6–8] or reported on a small number of deaths among women [9]. Furthermore, the majority of the studies used World Health Organization (WHO) or the Centers for Disease Control (CDC) classifications which include a broad normal BMI range as a reference category and consequently may attenuate the risk attributed to obesity and overestimate the threshold BMI associated with increased risk for death. Notably, the risk among underweight women and men at adolescence is controversial and it is unclear if the association is confounded by coexisting chronic illness [10, 11], or biased by recalled (vs. measured) BMI data [12]. We recently studied the association between BMI in late adolescence and future risk for death attributed to cardiovascular disease [13] or diabetes [14] in a cohort of 2.3 million adolescents. Here, we compared the sex-specific relationships between adolescent BMI and cardiovascular and non-cardiovascular mortality in midlife. Furthermore, we aimed at identifying and comparing the sex-specific BMI threshold values associated with increased risk for future mortality.

## Materials and methods

### Study population

**Fig. 1:**
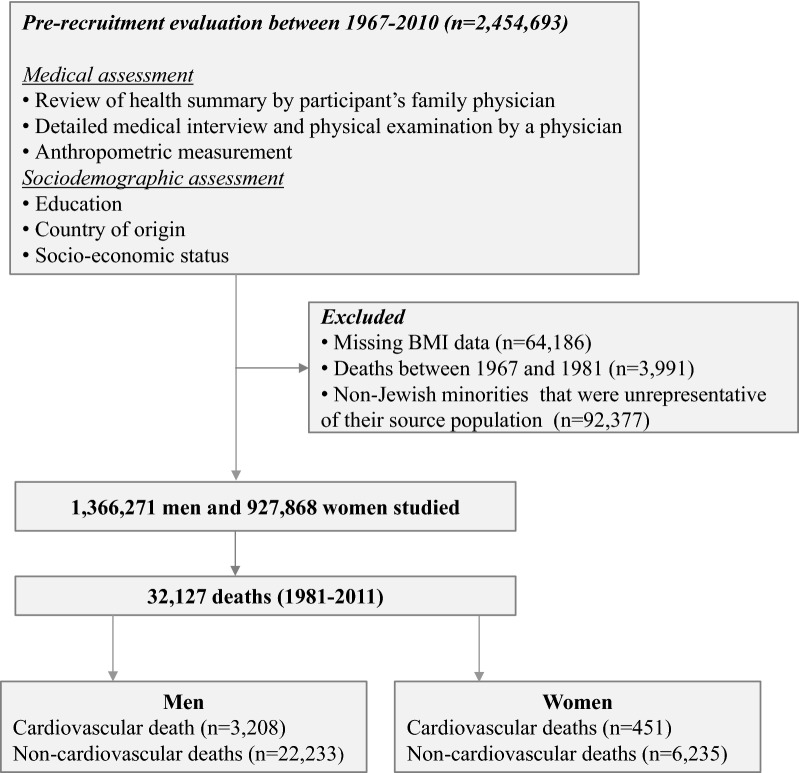
Flow diagram of study design and outcomes. Cardiovascular deaths were considered as those attributed to an underlying cardiovascular cause (ICD-9: 390–459; ICD-10: I00–I99) or diabetes (ICD-9: 250; ICD-10: E08–E13), whereas the remaining deaths were classified as non-cardiovascular. There were 1662 deaths (4.9% of the total N of deaths) for whom the cause of death was not available

All citizens obligated for military service in Israel are required to undergo a compulsory medical assessment at age of 17 years. Figure 1 displays the examination process and study design. Between January 1 1967 and December 31 2010 2,454,693 adolescents were examined at ages 16–19 years. Participants with missing BMI data (n = 64,186) and non-Jewish minorities who were unrepresentative of their source population (n = 92,377) were excluded from the analyses. Included in the final sample were 927,868 women and 1,366,271 men for a total of 2,294,139 examinees. As noted in previous studies of this cohort [13, 14], orthodox and ultra-orthodox Jewish women are not legally obligated for military service and therefore, may not be examined, and therefore may be under-represented here. The Jewish adolescent men in this study can be considered a nationally representative sample [13]. The Israeli Medical Corps Institutional Review Board provided ethical approval for the study and waived the need of informed consent given strict maintenance of participants’ anonymity (Fig. 1).

### Primary outcomes and documentation of the cause of death

The primary outcomes of the study were either death attributed to cardiovascular causes according to the International Classification of Diseases (ICD-9: 390–459; ICD-10: I00–I99) or deaths attributed to non-cardiovascular etiologies. Given that deaths attributed to diabetes (ICD-9: 250; ICD-10: E08–E13) are closely associated with cardiovascular deaths [15], we grouped these together with cardiovascular deaths as was performed in previous studies [16–18]. The underlying cause of death, as officially coded from death notifications by the Israel Central Bureau of Statistics, was linked to the database using the participants’ national ID number. The cause of death was available to us only from 1981 onwards. Deaths among recruits are documented in IDF computer records since 1967 with an indicator whether service-related (no other causes are provided). Follow-up terminated on June 30th 2011, or at the date of death, whichever came first.

### Data collection and study variables

Health examinations and review of the medical history were performed by trained military physicians. Standardized measurements of weight and height were undertaken by trained personnel with each participant barefoot and in underwear; BMI was calculated. Data regarding education, residential socioeconomic status, country of birth and country of origin were recorded. Education was grouped into ≤ 9, 10, 11 or 12 years of formal schooling. Socioeconomic status (SES), based on locality of residence at the time of study enrolment [19], was grouped into low, medium and high. Country of origin (classified by father’s or grandfather’s country of birth if the father was born abroad) and country of birth were grouped as reported previously [13].

### Statistical analysis

BMI was treated as a continuous variable and was also grouped according to a modification of the World Health Organization classification by splitting of the normal range, with the following subgroups: BMI < 18.50 (underweight), 18.50 ≤ BMI < 22.0 (low-normal), 22.0 ≤ BMI < 25.0 (high-normal), 25.0 ≤ BMI < 30.0 (overweight) and BMI ≥ 30 kg/m^2^ (obese), as at age 17 years adolescents have completed > 98% of their growth [20]. Person-year mortality rates were calculated with follow-up commencing from 1981. Cox proportional hazard models stratified by sex were used to estimate the hazard ratios (HRs) and 95% confidence intervals (CI) for CVD outcomes and non-CVD related outcomes with the low-normal BMI group as the reference category. We considered as potential confounders in the multivariable model all available variables that were significantly associated with BMI and cardiovascular outcomes (p < 0.05; age, birth year, sex, residential SES, education, country of origin and height). Adjusted Cox regression spline models (SAS, version 9.4) were fit to estimate the BMI value associated with minimum mortality risk for each of the study outcomes. Cubic splines with three equally spaced knots positioned between the minimum and maximum values of the variable were presented. Spline models for Cox proportional hazard were performed with SAS/STAT and SAS/GRAPH software version 9.4 SAS institute Inc., Cary, NC, USA. Sex interaction was computed with BMI as a continuous variable both in unadjusted and multivariable-adjusted models.

Several sensitivity analyses were conducted. We restricted the Cox analysis to those with unimpaired health status at baseline (i.e. no indication of any medical diagnosis in the medical review that would require chronic medical treatment or would limit ability to serve in a combat unit) [13, 21] to avert the possibility of reverse causality. In a separate analysis, we limited the analysis to participants with a follow-up of at least 3 decades (enrolled between 1967 and 1981) to allow a meaningful and equal period of follow-up between the sexes. We also analyzed the association between adolescent BMI and mortality with the reference group defined as the standard normal range (18.5–25.0 kg/m^2^). Multiple imputation was applied to those with missing data (1.4% of examinees) as reported previously [13]. Analyses were performed using SPSS (version 23.0), unless mentioned otherwise.

## Results

Baseline characteristics are shown in Table 1. The mean age at enrolment was 17.3 ± 0.4 years for women and 17.4 ± 0.4 years for men, with over 85% of inductees enrolled at age 17. The sample was heterogeneous as to the country of origin. The mean BMI values at baseline were 21.7 and 21.6 kg/m^2^ for women and men, respectively.

**Table 1:** Baseline characteristics of the study cohort

	BMI (kg/m^2^)					Total
	< 18.5	18.50–21.99	22.0–24.99	25.0–29.99	≥ 30.0	
*Women*						
Number of participants	128,876	448,712	224,937	99,867	25,476	927,868
Age (years)	17.3	17.3	17.3	17.3	17.3	17.3
Height (cm)	162.8	162.1	161.8	161.8	162.3	162.1
Mean BMI (kg/m^2^)	17.5	20.3	23.3	26.8	32.9	21.7
12 years schooling (%)	91	91	90	89	90	91
Low residential SES (%)	20	20	21	22	23	21
Born in Israel (%)	87	87	86	87	87	87
Country of origin (%)						
Israel	13.7	48.9	23.9	10.7	2.8	
USSR	13.7	47.3	24.5	11.4	3.1	
Asia	16.4	49.0	22.6	9.7	2.4	
Africa	12.5	47.1	25.2	12.0	3.3	
Europe	12.7	49.2	25.0	10.6	2.5	
Ethiopia	29.3	45.4	16.5	7.1	1.7	
*Men*						
Number of participants	186,190	678,005	324,665	139,556	37,855	1,366,271
Age (years)	17.3	17.3	17.4	17.4	17.4	17.4
Height (cm)	173.2	173.4	173.7	174.0	174.0	173.5
Mean BMI (kg/m^2^)	17.5	20.3	23.3	26.8	32.9	21.6
12 years schooling (%)	71	72	75	76	76	73
Low residential SES (%)	27	27	26	27	30	27
Born in Israel (%)	84	83	82	83	84	83
Country of origin (%)						
Israel	13.2	48.6	24.1	10.9	3.2	
USSR	11.2	46.0	26.4	12.7	3.7	
Asia	16.9	50.7	21.4	8.7	2.3	
Africa	12.7	51.6	23.5	9.5	2.7	
Europe	11.9	48.6	25.5	11.2	2.8	
Ethiopia	31.2	52.6	11.2	3.9	1.1	

During 17,346,230 and 28,367,431 person-years of follow up among women and men, respectively, there were 451 and 3208 CVD deaths (mean ages at death 41.9 ± 10.9 years and 47.0 ± 9.1 years), respectively, and 6235 and 22,233 non-CVD deaths (mean ages at death 39.3 ± 11.8 years and 37.5 ± 12.5 years), respectively. Notably, there were sex differences in the proportions of cardiovascular mortality; coronary heart disease, 21.3% in women vs. 43.7% in men; and stroke, 21.3% vs. 13.1%, respectively (Additional file 1: Table S1). The overall median follow-up of women and men was 17.4 years (intra-quartile range [IQR], 9.3–27.0) and 19.4 years (intra-quartile range 10.4–30.8), respectively. Obese adolescents had a shorter follow-up in both women (11.9, IQR 6.0–19.5) and men (11.5, IQR 5.9–20.1), reflecting the rise in prevalence of overweight and obesity in more recent years [22]. Table 2 shows person-year incidence rates and hazard ratios (HRs) for death among women and men, the latter both unadjusted and multivariable-adjusted. CVD mortality in men showed a fivefold excess across the BMI categories and total mortality a twofold excess compared with women. The underweight group was associated with the lowest rates of cardiovascular death among women and men, and was associated with an unadjusted (cardiovascular protective) HR compared to low-normal BMI in women of 0.68 (95% CI 0.46–0.98, p = 0.04). This finding persisted when the study sample was limited to women with unimpaired health (347 deaths; HR = 0.63, 95% CI 0.40–1.00, p = 0.049; Additional file 1: Table S2), and was accentuated when the standard normal range (18.5–24.9 kg/m^2^) was set as the reference category (HR = 0.59, 95% CI 0.41–0.86, p = 0.006). However, the strength of the association among obese women was reduced. Limiting the study sample to participants with at least 3 decades of follow-up was associated with an equal duration of follow-up between the sexes (mean for men, 36.9 ± 5.2 years; women 36.8 ± 4.5 years), reduced the sex differences in the age of death and accentuated the findings (Additional file 1: Table S3). Both women and men exhibited increased risk for cardiovascular mortality among the high-normal BMI group [HR of 1.42 (95% CI 1.14–1.77) vs. 1.53 (95% CI 1.40–1.67), respectively], overweight [2.13 (95% CI 1.63–2.78) vs. 2.99 (95% CI 2.71–3.31), respectively] and obese adolescents [3.90 (95% CI 2.47–6.14) vs. 5.40 (95% CI 4.60–6.33), respectively] compared with low-normal BMI (18.50–21.99 kg/m^2^) group. Nevertheless, there was no statistically significant interaction between sex and BMI for CVD death (p for interaction = 0.62).

**Table 2:** Hazard ratios for CVD and non-CVD mortality in women and men stratified by BMI categories

	BMI (kg/m^2^)					Total or BMI as a continuous variable
	< 18.5	18.50–21.99	22.0–24.99	25.0–29.99	≥ 30.0	
*Women*						
N of participants	128,876	448,712	224,937	99,867	25,476	927,868
Median follow-up (25th; 75th)	16.4 (10.0, 25.6)	18.0 (9.7, 27.9)	17.9 (9.6, 27.9)	15.9 (8.3, 24.8)	11.9 (6.0, 19.5)	17.4 (9.3, 27.0)
Cumulative follow-up (person-years)	2,301,449	8,641,690	4,305,855	1,745,031	352,203	17,346,230
*Cardiovascular mortality (451 deaths)*						
N of deaths	31	189	134	76	21	451
Crude incidence (per 10^5^ person years)	1.34	2.18	3.11	4.35	5.96	2.59
HR	0.68	1 (ref)	1.42	2.13	3.90	1.12 3
95% CI	0.46–0.98		1.14–1.77	1.63–2.78	2.47–6.14	1.096–1.151
p	0.043		0.002	2.9*10^−8^	4.4*10^−9^	3.3*10^−20^
*Non-cardiovascular mortality (6235 deaths)*						
N of deaths	741	3008	1621	707	158	6235
Crude incidence	32.19	34.80	37.64	40.51	44.86	35.94
HR	1.01	1 (ref)	1.07	1.23	1.66	1.031
95% CI	0.93–1.10		1.01–1.14	1.13–1.33	1.42–1.95	1.02–1.04
P	0.80		0.021	10^−6^	5.3*10^−10^	4.8*10^−14^
*Men*						
N of participants	186,190	678,005	324,665	139,556	37,855	1,366,271
Median follow-up (25th; 75th)	19.4 (10.9, 29.8)	20.4 (11.3, 31.9)	19.3 (10.1, 31.1)	16.1 (8.1, 26.9)	11.5 (5.9, 20.1)	19.4 (10.4, 30.8)
Cumulative follow-up (person-years)	3,834,407	14,685,322	6,744,963	2,555,251	547,485	28,367,431
*Cardiovascular mortality (3208 deaths)*						
N of deaths	306	1284	865	578	175	3208
Crude incidence (per 10^5^ person years)	7.98	8.74	12.82	22.62	31.96	11.30
HR	0.99	1 (ref)	1.53	2.99	5.40	1.14
95% CI	0.88–1.13		1.40–1.67	2.71–3.31	4.60–6.33	1.13–1.15
p	0.93		8.7*10^−22^	10^−103^	1.8*10^−95^	3.7*10^−169^
*Non-cardiovascular mortality (22,233 deaths)*						
N of deaths	2970	11,184	5358	2158	563	22,233
Crude incidence (per 10^5^ person years)	77.45	76.15	79.43	84.45	102.83	78.37
HR	1.04	1 (ref)	1.06	1.16	1.48	1.019
95% CI	1.00–1.09		1.03–1.10	1.11–1.21	1.36–1.61	1.01–1.02
p	0.040		0.0003	6.1*10^−10^	1.8*10^−19^	2.6*10^−17^

The association was assessed with Cox models adjusted for age, sex, birth year, residential socio-economic status, education, country of origin, and height

**Fig. 2:**
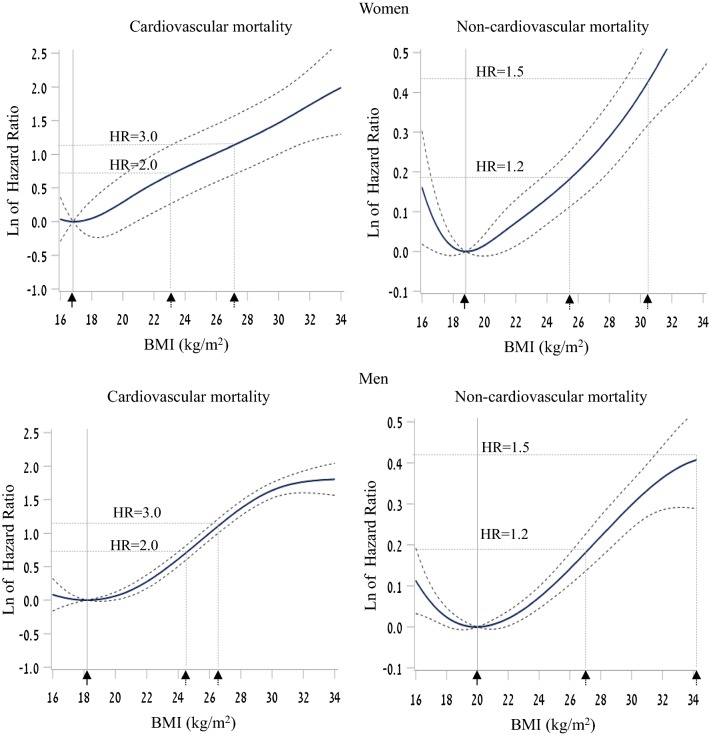
The relationship between adolescent BMI and mortality. Spline analysis demonstrating the non-linear relationship between BMI at adolescence and adulthood mortality among women (upper panels) and men (lower panels) comparing cardiovascular disease-related mortality (left) and non-cardiovascular disease mortality (right). The Cox models were adjusted for age, birth year, sex, residential socioeconomic status, education, country of origin and height. Dashed lines show Hazard ratios of 1.2, 1.5, 2.0 and 3.0 and their matching BMI levels (dashed arrows). The BMI level where minimal risk exists is marked by a vertical line and a full arrow

Underweight women did not exhibit excess risk for non-CVD death, whereas underweight men had a small, but significant, increased adjusted risk (HR = 1.04; 95% CI = 1.00–1.09, p = 0.04). For this outcome, an increased risk was observed for the high-normal, overweight and obese groups in both sexes with an interaction between sex and BMI in an unadjusted model (p for interaction = 0.012) reflecting the stronger associations among women, that became less significant in the multivariable model (p for interaction = 0.069). Multivariable spline models indicated a minimum risk for cardiovascular death at adolescent BMI values of 16.8 and 18.8 kg/m^2^ for women and men, respectively (corresponding to the 3rd and 13th BMI US-CDC percentiles for men and women aged 17.4 years, respectively; Fig. 2). The threshold adolescent BMI values for a significantly increased cardiovascular mortality risk were 21.0 and 21.4 kg/m^2^ for women and men, respectively. For non-CVD death, the minimum risk BMI values computed were 18. 2 and 20.0 kg/m^2^ for women and men, respectively (corresponding to the 12th and 29th BMI US-CDC percentiles for men and women aged 17.4 years, respectively), with significantly increased non-CVD mortality risk at BMI values of 20.0 and 22.0 kg/m^2^, respectively.

## Discussion

Our study presents sex-specific associations of adolescent BMI with CVD and non-CVD mortality by mid-adulthood in a national cohort of young Israeli women and men. The main findings of the study are that underweight was protective for CVD mortality among adolescent women, but was associated with excess all-cause mortality among men. The optimal BMI was lower in women than in men; 16.8 kg/m^2^ vs. 18.8 kg/m^2^ for CVD mortality, and 18.2 kg/m^2^ vs. 20.0 kg/m^2^ for non-CVD mortality. There was a significant interaction between sex and BMI for non-CVD death. The association between BMI and non-CVD death was stronger for women than men in all groups except for underweight.

CVD mortality has declined substantially over the past several decades [23, 24], but there is evidence indicating that this positive trend among women is lagging behind that of men [25], especially at young age, and even some evidence for increased CVD mortality in young women [26, 27]. Our finding of an overall similar association between adolescent BMI and CVD mortality in both sexes is in agreement with previous reports [5, 28]. We report that overweight and obesity at adolescence were associated with an increased risk for cardiovascular death with lower risk estimates for cardiovascular mortality among women than men. Most previous studies showed a non-linear, ‘U’ or ‘J’-curve shaped, relationship between BMI and mortality [29–31]. Baker et al. found linear associations between childhood BMI and coronary heart disease with a mildly stronger association among boys [5]. In a study that included 238 overweight or obese adolescents that were followed for 60 years, Must et al. reported a HR for coronary mortality of 2.3 (1.4–4.1) and 13.2 (1.6–108.0) for overweight and obese boys, but no risk for overweight and obese girls [9]. While this finding was reported elsewhere [32],a large Norwegian study that included 230,000 adolescents that were followed for approximately 3 decades, showed a similar HR for cardiovascular death in both sexes [33]. These controversies may be attributed to differences in follow-up, definition of the reference group, exclusion of participants with BMI in the low normal range [9], or the definition used for cardiovascular mortality. There are conflicting results on the association between underweight and mortality risk [12, 31, 34, 35], with reports of excess mortality [30, 36–38] or reduced risk (only among women) [11], whereas in other studies the entire underweight group was excluded from analysis [9, 39]. Several methodological inconsistencies may contribute to the controversy. A wide age range at enrollment that can span over four [30, 37] or even seven decades [35] may blunt a strong association reported at a young age [4, 40]. Lack of systematic evaluation of health status at baseline [31, 34, 36] and use of recalled vs. measured BMI data [35, 38] are additional potential problems that were shown to particularly influence the underweight group [12]. In this regard, our study sample was homogenous in age of enrollment, included systematic evaluation of health status at baseline and was based exclusively on measured weight and height data. Additionally, a sensitivity analysis that was limited to participants with unimpaired health accentuated the lower risk for both CVD and non-CVD death among underweight women, but not among men.

The definition of abnormal BMI in childhood has garnered considerable attention due to the rise of obesity epidemic [41]. Yet, only a few studies have revisited the entire so-called normal BMI range and suggested sex-specific optimal BMI values in childhood with respect to fatal outcomes in adulthood. Here, the optimal BMI values for what appear to be well below the upper underweight cutoff for women and within the low normal range for men (note that the 5th BMI CDC percentile at age 17.4 years is 17.3 and 17.9 kg/m^2^ for women and men, respectively). Data from large meta-analyses that were limited to adults (with age at enrolment that spanned at least 4 decades) pointed to an optimal range between 23.0 and 25.0 kg/m^2^ for all-cause mortality, and 22.5 kg/m^2^ for cardiovascular mortality [28, 35, 37]. In an Austrian cohort that investigated the association of age specific BMI and all-cause mortality a clear trend of increasing optimal BMI was witnessed but children and adolescence were not included [42]. A pooled analysis of 239 studies indicated an optimal BMI value lower than 22.0 kg/m^2^ for all-cause mortality when the sample was limited to young adults (age 35–49 years) with significantly higher risk estimates for underweight men than underweight women [40].

Several lines of evidence support associations between underweight and metabolic fitness or longevity. Caloric restriction, defined as a reduced intake of calories not causing malnutrition, has been demonstrated as an intervention that can prolong life and health span [43] by activating cellular pathways such as autophagy [44, 45]. Epidemiological studies on lifespan of Okinawans in Japan reported low caloric intake, lower mean BMI, lower cardiovascular- and age-related morbidity and prolonged longevity with an accentuated association between lower BMI and cardiovascular morbidity among women than men in this population [46]. We have shown recently in this cohort that healthy underweight women, but not underweight men of any other BMI group, exceed by up to 4.1 cm their expected height at age 17 years, a well-established risk marker for cardiovascular health [47]. The mechanisms underlying these sex-specific differences are complex and may include sex hormones, gut microbiome-related mechanisms, nutrition and stress [48]. Additional link between lower BMI and long-term outcomes, most notably cardiovascular, was demonstrated following bariatric surgery, showing that both diabetic and non-diabetic patients enjoy a dramatic reduction in the risk for cardiovascular mortality decades following surgery [49].

Several limitations of the current study should be addressed. First, BMI data were collected at a single time point with no available adulthood BMI measurements, preventing us from determining whether the association presented here is independent of adulthood BMI. This is a limitation as there is evidence to support that trajectory of BMI from childhood to adulthood has affects outcomes [50, 51]. In a sub-sample of this cohort the correlation of adolescent BMI at age 17 years and adult BMI at age 50 years was 0.53 (i.e. an R^2^ of about 25%) [52]. This would be expected to dilute the association of adolescent BMI with mortality if all the association was mediated through adult BMI. Nevertheless, we find a substantive point estimate of the hazard ratio with lower 95% confidence bounds of 1.6 and 2.5 for overweight and obese women, respectively, suggesting BMI at adolescence to be a remarkably strong risk marker for fatal outcomes in midlife. Second, we were unable to adjust the reported results for established risk factors such as smoking and exercise level, and could also not adjust for the effect of adverse events or comorbidities which evolved from adolescence to early adulthood and may carry increased risk of adverse outcomes [53] as these were not collected in our cohort at age 17 years. Yet, the association remained nearly unchanged following adjustment for socio-demographic variables, which were previously shown to be strongly related to smoking in a subgroup of this cohort [54]. Third, other than BMI and its components, this cohort did not include other anthropometric measurements that were found to be strong predictors of CVD mortality, independent of BMI [55]. Nevertheless, BMI is a well-studied measure that is currently recommended by the US Preventive Services Task Force as the screening measure of choice for childhood and adolescent obesity [56]. In addition, as mentioned, we were unable to allocate the specific cause of death for individuals who died during the years 1967–1981. Nevertheless, we previously showed by simulations that this gap of knowledge is unlikely to change the association between adolescent BMI and cardiovascular-specific mortality. Finally, conclusions from this cohort are based on midlife mortality in which cardiovascular death constituted 11.2%, similarly to a fraction of 15% that was reported in a nationwide Swedish study whose design was similar to ours [57], and should not be extrapolated to older ages.

The strengths of the study include its large sample size, systematic collection of measured anthropometric data, long follow up and large person-years database. This allowed the analysis to focus specifically on women, with adequate power despite a relatively low rate of events in women in midlife.

## Conclusions

To conclude, underweight at adolescence appears to engender a protective effect among women but not men with respect to adulthood CVD-related mortality. The lowest risk for CVD related mortality is seen with lower than normal BMI values in women and in the low-normal BMI range in men.

## Additional file

**Additional file 1.** Additional tables.
