# Supplementary material for: Sex-specific associations between adolescent categories of BMI with cardiovascular and non-cardiovascular mortality in midlife
Source: Cardiovasc Diabetol. 2018 Jun 5;17:80. doi: 10.1186/s12933-018-0727-7 (PMC5989357; doi:10.1186/s12933-018-0727-7)
Supplement: Supplementary file 1 — Additional file 1. Additional tables. [file 12933_2018_727_MOESM1_ESM.docx]

**Table S1** – **Cardiovascular-specific cause of death stratified by sex**

|  | **BMI (kg/m^2^)** | | | | | **Total** |
| --- | --- | --- | --- | --- | --- | --- |
|  | **<18.5** | **18.50-21.99** | **22.0-24.99** | **25.0-29.99** | **≥30** |  |
| **Women** | | | | | | |
| Cerebrovascular disease | 22.6% | 29.6% | 25.4% | 17.1% | 0.0% | 24.4% |
| Coronary heart disease | 16.1% | 20.6% | 21.6% | 21.1% | 33.3% | 21.3% |
| Diabetes mellitus | 6.5% | 5.8% | 11.2% | 21.1% | 23.8% | 10.9% |
| Fatal arrhythmias | 6.5% | 12.7% | 9.7% | 5.3% | 19.0% | 10.4% |
| Arterial disease | 9.7% | 3.2% | 2.2% | 7.9% | 0.0% | 4.0% |
| Hypertensive disease | 9.7% | 3.2% | 1.5% | 2.6% | 0.0% | 2.9% |
| Pulmonary embolism | 3.2% | 6.3% | 6.0% | 7.9% | 0.0% | 6.0% |
| Cardiomyopathies | 3.2% | 3.7% | 3.7% | 0.0% | 9.5% | 3.3% |
| Heart failure | 3.2% | 0.5% | 5.2% | 1.3% | 0.0% | 2.2% |
| Others | 19.4% | 14.3% | 13.4% | 15.8% | 14.3% | 14.6% |
| **Men** | | | | | | |
| Cerebrovascular disease | 18.0% | 14.1% | 12.1% | 11.1% | 8.0% | 13.1% |
| Coronary heart disease | 37.6% | 45.5% | 44.3% | 43.8% | 37.7% | 43.7% |
| Diabetes mellitus | 9.5% | 10.0% | 12.6% | 19.9% | 28.6% | 13.5% |
| Fatal arrhythmias | 7.5% | 9.6% | 9.0% | 6.4% | 6.3% | 8.5% |
| Arterial disease | 3.9% | 3.3% | 2.8% | 2.4% | 1.7% | 3.0% |
| Hypertensive disease | 3.9% | 2.6% | 3.7% | 4.2% | 4.6% | 3.4% |
| Pulmonary embolism | 2.0% | 1.5% | 1.3% | 0.7% | 1.7% | 1.3% |
| Cardiomyopathies | 3.9% | 3.3% | 3.4% | 3.3% | 2.3% | 3.3% |
| Heart failure | 4.2% | 2.6% | 2.2% | 2.2% | 2.9% | 2.6% |
| Others | 9.5% | 7.5% | 8.7% | 6.1% | 6.3% | 7.7% |

Table S2 – **Hazard ratios for CVD and non-CVD mortality in women and men stratified by BMI categories in participants with unimpaired health**. Cox models were multivariable-adjusted as described in Table 2. Risk estimate of the main analysis (table 2) were included to facilitate comparison.

|  | **BMI (kg/m^2^)** | | | | |
| --- | --- | --- | --- | --- | --- |
|  | **<18.5** | **18.50-21.99** | **22.0- 24.99** | **25.0-29.99** | ≥**30.0** |
| **Women** | | | | | |
| **Cardiovascular mortality (347 deaths out of 684880 participants)** | | | | | |
| HR | 0.63 | 1 (ref) | 1.36 | 2.07 | 2.45 |
| 95%CI | 0.40-1.00 |  | 1.06-1.74 | 1.52-2.80 | 1.15-5.24 |
| p | 0.049 |  | 0.015 | 2.9 * 10^-6^ | 0.021 |
| HR (main analysis) | 0.68 | 1 (ref) | 1.42 | 2.13 | 3.90 |
| 95%CI | 0.46-0.98 |  | 1.14-1.77 | 1.63-2.78 | 2.47-6.14 |
| p | 0.043 |  | 0.002 | 2.9*10^-8^ | 4.4*10^-9^ |
| **Non-Cardiovascular mortality (4846 deaths out of 684880 participants)** | | | | | |
| HR | 0.95 | 1 (ref) | 1.07 | 1.21 | 1.32 |
| 95%CI | 0.87-1.05 |  | 1.00-1.15 | 1.10-1.33 | 1.04-1.69 |
| p | 0.33 |  | 0.04 | 0.0001 | 0.02 |
| HR (main analysis) | 1.01 | 1 (ref) | 1.07 | 1.23 | 1.66 |
| 95%CI | 0.93-1.10 |  | 1.01-1.14 | 1.13-1.33 | 1.42-1.95 |
| P | 0.80 |  | 0.021 | 10^-6^ | 5.3*10^-10^ |
| **Men** | | | | | |
| **Cardiovascular mortality (2280 deaths out of 981238 participants)** | | | | | |
| HR | 0.92 | 1 (ref) | 1.49 | 3.02 | 5.50 |
| 95%CI | 0.79-1.09 |  | 1.35-1.65 | 2.69 -3.39 | 4.43-6.84 |
| p | 0.33 |  | 7.5* 10^-15^ | 1.3 * 10^-76^ | 1.9 * 10^-53^ |
| HR (main analysis) | 0.99 | 1 (ref) | 1.53 | 2.99 | 5.40 |
| 95%CI | 0.88-1.13 |  | 1.40-1.67 | 2.71-3.31 | 4.60-6.33 |
| p | 0.93 |  | 8.7*10^-22^ | 10^-103^ | 1.8*10^-95^ |
| **Non-Cardiovascular mortality (16527 deaths out of 981238 participants)** | | | | | |
| HR | 1.04 | 1 (ref) | 1.07 | 1.19 | 1.52 |
| 95%CI | 0.99-1.10 |  | 1.03-1.11 | 1.13-1.25 | 1.35-1.70 |
| p | 0.097 |  | 0.001 | 3.8 * 10^-10^ | 1.0 * 10^-12^ |
| HR (main analysis) | 1.04 | 1 (ref) | 1.06 | 1.16 | 1.48 |
| 95%CI | 1.00-1.09 |  | 1.03-1.10 | 1.11-1.21 | 1.36-1.61 |
| p | 0.040 |  | 0.0003 | 6.1*10^-10^ | 1.8*10^-19^ |

The association was assessed with Cox models adjusted for age, sex, birth year, residential SES, education, country of origin, and height.

Table S3 – **Sensitivity analyses restricted to participants with at least 3 decades of follow-up.** Included in this sub-analysis were 181,002 and 370,943 women and men who were enrolled in the study between years 1967 and 1981. The mean follow-up for these participants was 36.94±5.18 and 36.78 ±4.53 years, respectively.

**(a) Hazard ratios for CVD and non-CVD mortality in women and men**. Models were multivariable-adjusted as described in Table 2. Risk estimates of the main analysis (Table 2) are presented to facilitate comparison.

|  | **BMI (kg/m^2^)** | | | | |
| --- | --- | --- | --- | --- | --- |
|  | **<18.5** | **18.50-21.99** | **22.0- 24.99** | **25.0-29.99** | ≥**30.0** |
| **Women** | | | | | |
| **Cardiovascular mortality (324 deaths out of 181002 participants)** | | | | | |
| HR | 0.62 | 1 (ref) | 1.40 | 2.32 | 4.25 |
| 95%CI | 0.39-0.99 |  | 1.08-1.82 | 1.71-3.16 | 2.40-7.52 |
| p | 0.034 |  | 0.011 | 8.6*10^-8^ | 6.9*10^-7^ |
| HR (main analysis) | 0.68 | 1 (ref) | 1.42 | 2.13 | 3.90 |
| 95%CI | 0.46-0.98 |  | 1.14-1.77 | 1.63-2.78 | 2.47-6.14 |
| p | 0.043 |  | 0.002 | 2.9*10^-8^ | 4.4*10^-9^ |
| **Non-Cardiovascular mortality (3873 deaths out of 181002 participants )** | | | | | |
| HR | 0.95 | 1 (ref) | 1.14 | 1.25 | 1.91 |
| 95%CI | 0.85-1.05 |  | 1.06-1.23 | 1.13-1.39 | 1.52-2.40 |
| p | 0.32 |  | 0.32 | 3*10^-6^ | 2.9*10^-8^ |
| HR (main analysis) | 1.01 | 1 (ref) | 1.07 | 1.23 | 1.66 |
| 95%CI | 0.93-1.10 |  | 1.01-1.14 | 1.13-1.33 | 1.42-1.95 |
| P | 0.80 |  | 0.021 | 10^-6^ | 5.3*10^-10^ |
| **Men** | | | | | |
| **Cardiovascular mortality (2,154 deaths out of 370943 participants)** | | | | | |
| HR | 0.98 | 1 (ref) | 1.50 | 3.10 | 5.74 |
| 95%CI | 0.85-1.12 |  | 1.37-1.65 | 2.75-3.39 | 4.84-6.79 |
| p | 0.71 |  | 3.4*10^-18^ | 2.1*10^-96^ | 3.4*10^-91^ |
| HR (main analysis) | 0.99 | 1 (ref) | 1.53 | 2.99 | 5.40 |
| 95%CI | 0.88-1.13 |  | 1.40-1.67 | 2.71-3.31 | 4.60-6.33 |
| p | 0.93 |  | 8.7*10^-22^ | 10-^103^ | 1.8*10^-95^ |
| **Non-Cardiovascular mortality (13,829 deaths out of 370943 participants)** | | | | | |
| HR | 1.07 | 1 (ref) | 1.09 | 1.29 | 1.96 |
| 95%CI | 1.01-1.12 |  | 1.05-1.14 | 1.22-1.37 | 1.76-2.19 |
| p | 0.019 |  | 2.9*10^-5^ | 2.8*10^-17^ | 2.1*10^-32^ |
| HR (main analysis) | 1.04 | 1 (ref) | 1.06 | 1.16 | 1.48 |
| 95%CI | 1.00-1.09 |  | 1.03-1.10 | 1.11-1.21 | 1.36-1.61 |
| p | 0.040 |  | 0.0003 | 6.1*10^-10^ | 1.8*10^-19^ |

The association was assessed with Cox models adjusted for age, sex, birth year, residential SES, education, country of origin, and height.

**(b) Age of death attributed to a cardiovascular cause**

The mean age of death is shown for the entire cohort and for participants with at least 3 decades of follow-up. Numbers in parentheses denote the number of deaths in a given category.

|  | | Age of death attributed to a cardiovascular cause (years) | | | | |
| --- | --- | --- | --- | --- | --- | --- |
|  |  | Coronary heart Disease | Stroke | Diabetes | Other CVD causes | Total |
| Entire period | Men | 47.7±8.0 (1401) | 47.1±10.2  (418) | 50.7±6.2  (432) | 44.3±10.3  (957) | 47.0±9.1 |
|  | Women | 43.5±10.6  (96) | 41.8±10.2  (110) | 48.2±9.0  (49) | 39.6±11.0  (196) | 41.9±10.9 |
| Participants enrolled between 1967 and 1981 | Men | 48.9±7.0 (1278) | 49.0±8.7  (373) | 51.2±5.7  (414) | 47.2±8.4  (789) | 48.7±7.4 |
|  | Women | 46.2±9.3  (79) | 45.5±8.0  (77) | 50.9±7.3  (40) | 45.0±8.5  (128) | 46.9±8.4 |
